# Expression of putative targets of immunotherapy in acute myeloid leukemia and healthy tissues

**DOI:** 10.1038/leu.2014.14

**Published:** 2014-01-28

**Authors:** M Goswami, N Hensel, B D Smith, G T Prince, L Qin, H I Levitsky, S A Strickland, M Jagasia, B N Savani, J W Fraser, H Sadrzadeh, T Rajkhowa, S Ito, N A Jain, M Battiwalla, A T Fathi, M J Levis, A J Barrett, C S Hourigan

**Affiliations:** 1 Myeloid Malignancies Section, National Heart, Lung and Blood Institute, National Institutes of Health, Bethesda, MD, USA; 2 Hematology Branch, National Heart, Lung and Blood Institute, National Institutes of Health, Bethesda, MD, USA; 3 Johns Hopkins University School of Medicine, Baltimore, MD, USA; 4 Cancer Immunology Experimental Medicine, Pharma Research and Early Development, Roche Glycart AG, Schlieren, Switzerland; 5 Division of Hematology and Oncology, Vanderbilt University Medical Center, Nashville, TN, USA; 6 Center for Leukemia and the Bone Marrow Transplant Unit, Division of Hematology/Oncology, Massachusetts General Hospital, Harvard Medical School, Boston, MA, USA

The ability to target myeloid malignancies using immunotherapy through means other than allogeneic transplantation depends on the capability to target leukemic clones while sparing normal tissues. It is now possible to generate clinical grade *ex-vivo* expanded T cells specific for leukemia-associated antigens (LAAs) for use in adoptive cell therapy.^1^ Although a variety of putative LAAs in acute myeloid leukemia (AML) have been identified for use as potential targets for immunotherapy^2, 3, 4, 5, 6, 7, 8^ and consensus panels have attempted to prioritize generic cancer antigens,^9^ a comprehensive evidence-based list of AML antigen targets has not yet been established. As a first step toward this goal, we therefore analyzed, using quantitative real-time PCR, the gene expression of 65 potential LAAs (Supplementary Table S1) in de-identified, clinically annotated samples from 48 newly diagnosed untreated AML patients that were collected under institutional review board-approved protocols from three NCCN cancer centers.

A total of 52 samples (30 peripheral blood (PB) and 22 bone marrow aspirate (BM) samples) from 48 AML patients were analyzed, which included 4 patients for whom both PB and BM samples were available. The average age of the patients was 52 years (range 24–86); 52% of the patients were women. A total of 7 patients had favorable cytogenetics, whereas 11 were classified as adverse, 13 patients had FLT3 mutations (including 8 patients with FLT3-ITD) and 9 patients had mutations in NPM1 (Supplementary Tables S2 and S3). RNA and DNA were isolated from the ficoll-purified PB and BM samples using AllPrep Mini Kits (Qiagen, Valencia, CA, USA), and the quantity, quality and integrity of isolated RNA were assessed using a Nanodrop 1000 Spectrophotometer (Wilmington, DE, USA) and Agilent RNA 6000 Nano Kit and 2100 Bioanalyzer (Santa Clara, CA, USA). Only RNA with an RNA Integrity Number (RIN) of 7.0 or greater was used for subsequent analysis (Supplementary Table S4). An amount of 400 ng high-quality, total RNA was reverse-transcribed into cDNA using RT2 First Strand Kit (Qiagen). Custom RT2 Profiler PCR array plates (SABiosciences, Qiagen) were used for PCRs performed using RT2 SYBR Green ROX qPCR Mastermix (SABiosciences) according to the manufacturer’s instructions on an ABI 7900 thermal cycler (Applied Biosystems, Foster City, CA, USA) with a program of 10 min at 95°C, followed by 40 cycles at 95°C for 15 s and at 60°C for 1 min. Controls for human genomic DNA contamination, reverse transcription and PCR efficacy were included. Fold-change expression values were calculated according to the comparative C(t) method.^10^ ΔC(t) was calculated as the C(t) of target gene ‘*X*’ minus the geometric mean C(t) of reference genes *HPRT1, PPIH* and *TFRC* in a sample. ΔC(t) for each target gene ‘*X*n healthy donor samples was also computed in this manner. To calculate ΔΔC(t), median ΔC(t) of gene ‘*X*’ in healthy donor blood or BM (depending on the source of the AML sample) was subtracted from the ΔC(t) of X in the AML sample (ΔC(t) of X in AML sample−median ΔC(t) of X in healthy donor samples). Statistical analysis was performed using GraphPad Prism (La Jolla, CA, USA).

We observed considerable heterogeneity in levels of RNA overexpression (OE) of putative LAAs compared with healthy donor tissues (Figure 1). Every AML sample had at least one LAA overexpressed, but no antigen was overexpressed in any of the AML samples. Surprisingly, the hemoglobin gamma globin gene *HBG2*, ordinarily expressed in the fetal liver, spleen and BM but not usually in adulthood, which was recently identified as a leukemia antigen in a study of induced immune responses to GVAX/K562 vaccination in chronic myeloid leukemia (CML),^11^ was found to be frequently overexpressed in AML, often to a high level (Figures 1a and 2). Similarly, *CCNA1*, *WT1*, *BAALC*, *PR3* and *PRAME* were also highly overexpressed in multiple AML samples (Figure 2). We were able to confirm the previously reported^12^^13^ association between FLT3-ITD-mutated AML and OE of *WT1* (Supplementary Figure S2) but not of the other antigens. Finally, consistent with the fact that much of the existing evidence for myeloid LAA OE has been derived from the study of leukemic cell lines, we noted that the K562 human CML blast phase erythroleukemia cell line (ATCC, Manassas, VA, USA) served as an excellent positive control for our panel with high expression of multiple, previously described, putative LAAs including *RHAMM*, *Survivin*, *h-TERT*, *CA9/CAIX*, *MAGEA3/6*, *MAGEB2* and *MAGEC1* (Supplementary Figure S1B) that were rarely detectable in our primary samples from AML patients (Figure 1a).

The ideal targetable leukemia antigen would have high tumor-specific expression but no expression in healthy tissues.^2^ We therefore also quantified tissue expression of these putative LAAs in a range of normal tissues that included samples (10 PB and 7 BM) collected under institutional review board-approved protocols at the NIH clinical center from 17 healthy donors with an average age of 41, 70% of whom were male, with ficoll purification and RNA extraction as described above. In addition, purified total RNA from a wide panel of human organs was obtained from Clontech (Mountain View, CA, USA) (Supplementary Table S5). Expression in at least some forms of healthy tissues was observed for almost all putative antigen targets (Figure 1b).

Several technical features are worthy of note. We found that samples with RIN scores of less than 7.0 resulted in higher than expected C(t) outputs, which correspond to lower gene expression when compared with samples with higher RIN scores (Supplementary Figure S3) and were therefore excluded from analysis. Our array platform was highly sensitive and reproducible (Supplementary Figure S1A), allowing for the reliable medium throughput analysis performed here. In most cases, antigen expression profiles from presentation blood and marrow samples from the same patient correlated closely (Supplementary Figure S4). Gene expression of LAA in phenotypically identified AML blast populations sorted through flow cytometry did not markedly differ from the gene expression seen in the presentation PB sample from which they were isolated (Supplementary Methods; Supplementary Table S6). Finally, we were able to detect LAA OE across multiple samples from the same patient including unsorted PB samples, sorted AML blasts and a sorted (that is lineage negative, CD34 positive and CD38 negative) PB population enriched for stem cells (Supplementary Methods; Supplementary Table S6).

This work has several obvious limitations. We performed this work on ‘real world’ first-presentation primary samples from three different leukemia centers in an attempt to limit bias introduced by presentation and referral patterns. Nevertheless, all institutions are highly specialized tertiary academic medical centers located in the northeastern and southeastern United States. Although we do have patient demographics on age and gender (Supplementary Table S2) and all these samples were from the first diagnosis before initiation of treatment for AML, we unfortunately do not have any information on race or ethnic background, medical history including details on antecedent hematological conditions, prior/concurrent malignancies or current medications with epigenetic or immune activity. We quantified total RNA expression levels (necessary but not sufficient for a targetable LAA) but did not provide information on protein expression or epitope processing and presentation by major histocompatibility complex; these factors will be addressed in future work now that the list of candidate AML LAAs has been substantially refined to exclude those not overexpressed in AML. Neo-antigens (that is, those generated by somatic mutations including single nucleotide variations, insertions, deletions and splice variants) are an important potential class of AML LAAs that were not investigated in this work, although extensive data on these AML-specific sequence changes are now available and immune responses to epitopes created by these mutations have recently been described.^14^

The ideal AML LAA would be expressed in most or all cases of AML but not in healthy tissues. Using a novel, highly sensitive and reproducible, real-time reverse transcription–PCR array testing only high-quality RNA we show in this work that the majority of proposed ‘LAAs’ are expressed in the leukemia cell line K562 but often not in primary samples from AML patients. Although we identified no antigen that was universally overexpressed in all AML samples, every patient did have at least one potentially targetable antigen overexpressed. We also noted significant healthy organ-specific tissue expression of many LAAs, highlighting the possibility of ‘off-target’ effects, a finding not evident from the study of expression levels in PB and BM alone. This list of genes overexpressed in AML, together with information regarding expression in a wide range of healthy tissue types, may be of use in AML as a reference for the selection of antigenic targets in adoptive T-cell therapy and may also have use in the PCR-based detection of minimal residual disease.^15^

## Figures and Tables

**Figure 1:**
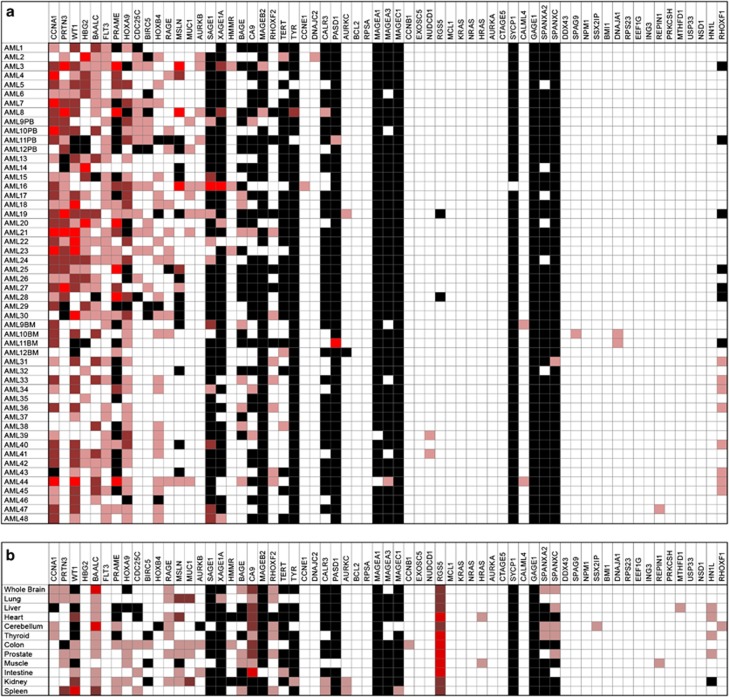
Expression of proposed leukemia associated antigens in acute myeloid leukemia (AML) patient samples and healthy tissues. (**a**) No single antigen was expressed in all cases of AML and many proposed antigen candidates are not frequently overexpressed in AML. BM, bone marrow; PB, peripheral blood. Fold change OE compared with median expression in healthy donors where light red indicates OE of 5–50 × , red indicates OE of 50–500 × , bright red indicates OE >500 × . Black indicates no detectable expression; white indicates expression values seen in similar range as healthy donors. First 30 AML samples listed were from PB and are therefore compared with healthy donor PB, the remaining 18 are from BM and are compared with expression in healthy donor BM. (**b**) Antigen expression in various human tissue types. Compared with median expression in healthy donors using same heat-map schema same as above.

**Figure 2:**
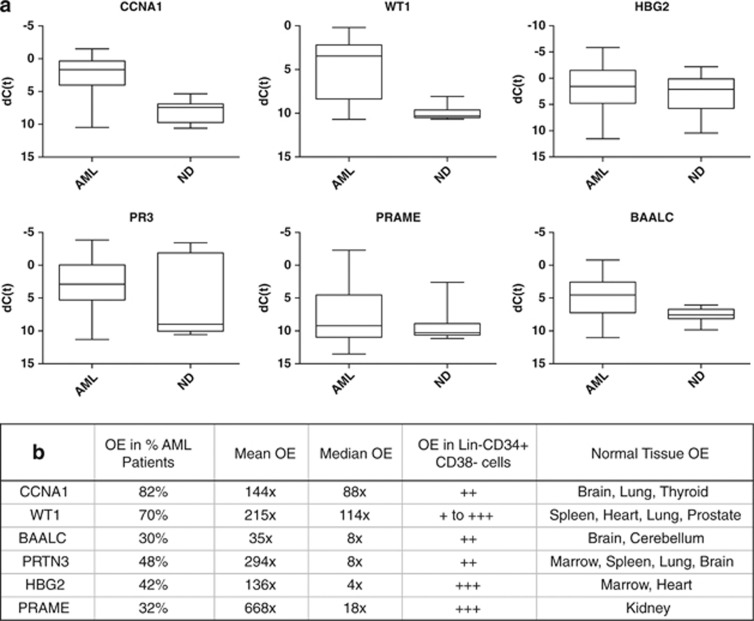
Top six acute myeloid leukemia-associated antigens. (**a**) Therapeutic or diagnostic threshold for leading antigen candidates. Expression of antigens in all AML samples compared with all normal donor samples as box-plots where box represents 25th–75th percentile and whiskers represent maximum and minimum dCT values observed. (**b**) Detailed characteristics of leading AML antigen candidates including what percentage of AML samples tested had at least 5 × OE of that gene, average levels of OE and OE in sorted lineage negative, CD34-positive and CD38-negative cells and normal donor tissues (see Supplementary Information for additional details).
