# Supplementary material for: Expression of putative targets of immunotherapy in acute myeloid leukemia and healthy tissues
Source: Leukemia. 2014 Jan 28;28(5):1167–70. doi: 10.1038/leu.2014.14 (PMC4013200; doi:10.1038/leu.2014.14)
Supplement: Supplementary Information [file leu201414x1.pdf]

Supplemental Table of Contents

Supplemental Methods. FACS of leukemic populations, RNA amplification, and qRT-PCR of sorted populations

Table S1. List of 65 known and potential acute myeloid leukemia-associated antigens.

Table S2. Clinical annotation of 48 AML patients.

Table S3. AML mutations in our AML population.

Table S4. RNA integrity numbers (RIN).

Table S5. Normal organ tissue information.

Table S6. Antigen expression in sorted leukemia cell populations of 8 AML patients.

Figure S1. Expression of leukemia-associated antigens (LAA) in K562.

Figure S2. Antigen expression in FLT3-ITD mutated AML.

Figure S3. RNA integrity numbers versus expression of common housekeeping genes.

Figure S4. Peripheral blood (PB) versus bone marrow (BM) from the same patient.

Supplemental Methods

Eight (8) AML PB samples were further characterized by sorting into leukemic blast, enriched stem cell, and leukemia-negative populations.

Up to 50 million cells from each of the 8 patients were stained with the following antibodies from Biolegend (San Diego, CA) with manufacturer's recommended concentration: APC CD13, PE CD33, PerCP/Cy5.5 CD34, BV 711 CD38, APC/Cy7 CD45, PE/Cy7 CD117, BV 421 CD123, and Alexa Fluor 700 HLA-DR in the dark at room temperature for 30 minutes.

Another set of up to 50 million cells from each of the 8 patients were stained with:PerCP/Cy5.5 CD34, BV 711 CD38, APC/Cy7 CD45RA, CD90, CD96, BV 421 CD123, PE TIM-3, and FITC Lineage (CD3, CD14, CD16, CD19, CD20, CD56) markers (Biolegend, San Diego, CA), and Alexa Fluor 647 CD96 (ABD Serotec, Raleigh, NC) in the dark at room temperature for 30 minutes.

Cells were then washed twice with cell staining buffer (Biolegend, San Diego, CA), suspended in 200uL cell staining buffer, and analyzed on the BD FACSaria III cell sorter (BD Biosciences, San Jose, CA). Leukemic blasts were identified based on available clinical markers, and a leukemia-negative population selected based on negativity of leukemic markers were sorted into cold RPMI 1600 with 40% FBS. Lineage negative (Lin-) CD34 positive CD38 negative cells were sorted into cold RPM1 1600 with 40% FBS.

Sorted cell populations were spun down at 1600rpm for 10 minutes and RNA and DNA was isolated using AllPrep Mini Kits (Qiagen, Valencia, CA) according to per manufacturer's protocol. Due to the low yield of RNA from these sorted populations, a pre-amplification step with primer mix (Qiagen, Valencia, CA) intended for use with our custom-designed arrays was performed before reverse transcription to amplify targets of interest according to manufacturer's protocol. qRT-PCR was performed according to the protocol described in the manuscript, and each population from each sample was run in triplicate. Fold change values were calculated using the comparative C(t) method using the geometric mean of 3 housekeeping genes that were expressed.

Table S1. List of 65 known and potential acute myeloid leukemia-associated antigens.

Antigen	Full Gene Name	UniGene	GenBank
WT1	Wilms tumor 1	Hs.591980	NM_000378
PRAME	Preferentially expressed antigen in melanoma	Hs.30743	NM_006115
HMMR (RHAMM)	Hyaluronan-mediated motility receptor	Hs.740467	NM_012484
PRTN3 (PR3)	Proteinase 3	Hs.928	NM_002777
BIRC5 (survivin)	Baculoviral IAP repeat containing 5	Hs.744872	NM_001168
TERT (hTERT)	Telomerase reverse transcriptase	Hs. 492203	NM_198253
TYR	Tyrosinase	Hs. 503555	NM_000372
BCL2	B-cell CLL/lymphoma 2	Hs. 150749	NM_000633
RPSA	Ribosmal protein SA	Hs. 449909	NM_002295
FLT3	Fms-related tyrosine kinase 3	Hs. 507590	NM_004119
CA9 (G250/CAIX)	Carbonic anhydrase IX	HS. 63287	NM_001216
MAGEA1	Melanoma antigen family A, 1	Hs. 72879	NM_004988
MAGEA3 (A6)	Melanoma antigen family A, 3	Hs. 417816	NM_005362
MAGEB2	Melanoma antigen family B, 2	Hs. 113824	NM_002364
MAGEC1	Melanoma antigen family C, 1	Hs. 132194	NM_005462
CCNA1	Cyclin A1	Hs. 417050	NM_003914
CCNB1	Cyclin B1	Hs. 23960	NM_031966
CCNE1	Cyclin E1	Hs. 244723	NM_001238
EXOSC5	Exosome component 5	Hs. 283741	NM_020158
NUDCD1	Nudc domain containing 1	Hs. 380291	NM_032869
RGS5	Regulator of G-protein signaling 5	Hs. 24950	NM_003617
MSLN	Mesothelin	Ns. 408488	NM_005823
DNAJC2 (MPP11)	DnaJ (HSP40) homolog, subfamily C, member 2	Hs. 558476	NM_014377
HOXA9	Homeobox A9	Hs. 659350	NM_152739
BAGE	B melanoma antigen	Hs. 545789	NM_001187
MCL1	Myeloid cell leukemia sequence 1	Hs. 632486	NM_021960
KRAS	V-Ki-ras22 Kirsten rat sarcoma viral oncogene homolog	Hs. 505033	NM_004985
NRAS	Neuroblastoma RAS viral (v-ras) oncogene homolog	Hs. 486502	NM_002524
HRAS	V-Ha-ras harvey rat sarcoma viral oncogene homolog	Hs. 37003	NM_005343
MUC1	Mucin 1, cell surface associated	Hs. 89603	NM_001018016
CALR3	Calreticulin 3	Hs. 304020	NM_145046
AURKA	Aurora kinase A	Hs. 250822	NM_003600
AURKB	Aurora kinase B	Hs. 442658	NM_004217
RAGE	Renal tumor antigen	Hs. 104119	NM_014226
CTAGE5	CTAGE family, member 5	Hs. 509200	NM_005930
SYCP1	Synaptonemal complex protein 1	Hs. 112743	NM_003176
CALML4	Calmodulin-like 4	Hs. 709550	NM_033429
SAGE1	Sarcoma antigen 1	Hs. 195292	NM_018666
XAGE1A	X antigen family, member 1A	Hs. 112208	NM_001097592
GAGE1	G antigen 1	Hs. 632815	NM_001468
SPANXA2	SPANX family, member A2	Hs. 334464	NM_145662
SPANXC	SPANX family, member C	Hs. 558533	NM_022661
DDX43 (HAGE)	DEAD (Asp-Glu-Ala-Asp) box polypeptide 43	Hs. 125507	NM_018665
SPAG9	Sperm associated antigen 9	Hs. 463439	NM_003971
NPM1	Nucleophosmin (nucleolar phosphoprotein B23, numatrin)	Hs. 557550	NM_199185
PASD1	PAS domain containing 1	Hs. 160594	NM_173493
BAALC	Brain and acute leukemia, cytoplasmic	Hs. 533446	NM_024812
SSX2IP	Synovial sarcoma, X breakpoint 2 interacting protein	Hs. 22587	NM_014021
HOXB4	Homeobox B4	Hs. 664706	NM_024015
BMI1	BMI1 polycomb ring finger oncogene	Hs. 380403	NM_005180
DNAJA1	DnaJ (Hsp40) homology, subfamily A, member 1	Hs. 445203	NM_001539
RPS23	Ribosomal protein S23	Hs. 144835	NM_001025
EEF1G	Eukaryotic translation elongation factor 1 gamma	Hs. 144835	NM_001404
CDC25C	Cell division cyle 25 homolog C (S. pombe)	Hs. 656	NM_001790
ING3	Inhibitor of growth family, member 3	Hs. 489811	NM_198267
HBG2	Hemoglobin, gamma G	Hs. 302145	NM_000184
REPIN1	Replication inhibitor 1	Hs. 647086	NM_013400
PRKCSH	Protein kinase C substrate 80K-H	Hs. 610830	NM_002743
MTHFD1	Methylenetetrahydrofolate deyhydrogenase (NADP+ dependent) 1	Hs. 652308	NM_005956
USP33	Ubiquitin specific peptidase 33	Hs. 480597	NM_201624
NSD1	Nuclear receptor binding SET domain protein 1	Hs. 106861	NM_022455
HN1L	Hematological and neurological expressed 1-like	Hs. 513261	NM_144570
RHOXF2	Rhox homeobox family, member 2	Hs. 567620	NM_032498
RHOXF1	Rhox homeobox family, member 1	Hs. 644617	NM_139282
AURKC	Aurora kinase C	Hs. 98338	NM_003160

Table S2. Clinical Annotation of 48 AML Patients.

Patient Code	PB or BM	Age	Sex	Cytogenetics Risk	Cytogenetic Details	FLT-ITD mutated	FLT3-TKD mutated	NPM1 mutated	IDH1 mutated	IDH1 mutated	Subtype	WBC @ Dx	%Blasts
AML1	PB	67	F	*Adverse*	"*complex" (2;12)*	N	N	N	N	N			
AML2	PB	39	M	Adverse	Complex	N	Yes	N	N	N	M1	2.5	54
AML3	PB	32	F	Favorable	8,21	N	N	N	N	N			
AML4	PB	34	F	Intermediate	8q24 [20]	N	N	N	N	N	M1	5.7	56
AML5	PB	51	F	Intermediate	47,XX,+21[1]/46,XX[19]	N	N	Yes	Yes	N	AML NOS	2.2	ND
AML6	PB	30	M	Intermediate	46XY [20]	N	N	N	N	N	M1	34.5	89
AML7	PB	39	M	Intermediate	Normal	Yes	N	N	N	N	M4	55.8	88
AML8	PB	32	F	Favorable	-X, t(8,21) [20]	N	N	N	N	N			
AML9PB	PB	64	F	Intermediate	46,XX[20]	N	N	N	N	N	M5	74.2	ND
AML10PB	PB	48	M	Favorable	46,XY,inv(16)(p13.1q22)[13]/46,XY[7]	N	N	N	N	N		24.6	81.9
AML11PB	PB	65	M	Intermediate	46XY [20]	N	N	Yes	N	N	M5	97.1	54.3
AML12PB	PB	60	M	Intermediate	46XY [20]	N	N	N	N	N			
AML13	PB	65	M	Adverse	45XY, -7 [15]	N	N	N	N	N			
AML14	PB	52	M	Intermediate	47XY, +21 [3]	N	N	N	N	N	M2	5.3	52
AML15	PB	58	F	Intermediate	46XX [20]	N	N	Yes	N	N			
AML16	PB	61	F	Intermediate	46XX [20]	N	N	N	N	N	M5		
AML17	PB	59	F	Intermediate	46XX [20]	N	N	N	N	N			
AML18	PB	44	F	Intermediate	47XX,+8[15]	Yes	N	N	N	N			
AML19	PB	70	F	Adverse	Complex	N	N	Yes	N	N			
AML20	PB	39	F	Favorable	t(8;21)	N	N	N	N	N	M2	5	35
AML21	PB	56	M	Favorable	46,XY,t(15;17)(q24;q21)[17]/46,XY[3]	N	N	N	N	N	M3	4.5	11.6
AML22	PB	40	F	Intermediate	Normal	Yes	N	Yes	N	N	M1	38.8	72
AML23	PB	36	F	Favorable	t(15;17)	Yes	N	N	N	N	M3	22.4	76
AML24	PB	31	M	Intermediate	t(6;9)	Yes	N	N	N	N	M2	7	47
AML25	PB	57	F	Intermediate	t(6:12:8)	N	N	N	N	N	M2	11.4	58
AML26	PB	31	F	Intermediate	Normal	N	N	N	N	N	M2	7.9	25
AML27	PB	51	F	Adverse	Complex	N	N	N	N	N	M5	19.5	52
AML28	PB	57	F	Intermediate	Normal	N	N	Yes	N	N	M5	61.9	73
AML29	PB	24	M	Intermediate	+21 [19]	N	N	N	N	N	M1	83.4	100
AML30	PB	55	F	Intermediate	Normal	Yes	N	N	N	N		1.1	63
AML31	BM	76	M	Intermediate	46XY [20]	N	N	N	N	N	MDS/AML		
AML32	BM	56	F	Adverse	-5q [72.5%]	N	N	N	N	N	MDS/AML		
AML33	BM	61	M	Intermediate	46XY [20]	N	N	N	N	Yes			
AML34	BM	60	M	Intermediate	46XY [20]	N	N	Yes	Yes	N	TherapyR	21.3	53
AML35	BM	56	F	Adverse	Complex	N	N	N	N	N			
AML36	BM	69	M	Intermediate	46XY [20]	N	N	N	N	Yes			
AML37	BM	68	M	Adverse	Complex	N	N	N	N	N			
AML38	BM	68	F	Intermediate	47XX,+8[15]	N	Yes	N	N	Yes			
AML9BM	BM	64	F	Intermediate	46,XX[20]	N	N	N	N	N	M5	74.2	89
AML10BM	BM	48	M	Favorable	46,XY,inv(16)(p13.1q22)[13]/46,XY[7]	N	N	N	N	N		24.6	99
AML11BM	BM	65	M	Intermediate	46XY [20]	N	N	Yes	N	N	M5	97.1	39
AML12BM	BM	60	M	Intermediate	46XY [20]	N	N	N	N	N			
AML39	BM	86	M	Intermediate	add(2), +8	N	N	Yes	N	N	Monocytic		78
AML40	BM	73	M	Intermediate	46,XY[cp4].nuc ish(ETV6x2)[100]	Yes	N	N	N	N	TherapyR		64
AML41	BM	68	M	Adverse	Complex	N	N	N	N	Yes	Monocytic		56
AML42	BM	42	M	Intermediate	46XY [20]	N	Yes	N	Yes	N	MultilinDysp		83
AML43	BM	78	M	Intermediate	46XY [20]	N	N	N	N	Yes	MDS/AML		40
AML44	BM	33	F	Favorable	Inv16	N	Yes	N	N	N			33
AML45	BM	67	F	Adverse	46XX,der(4:12)(q10;q10),del9(q13),del(11)(q13q23),+Mar[20]	N	N	N	N	N		1.1	2
AML46	BM	50	M	Adverse	47XY,+4,3,7dmin[3}/46,XY,1,15dmin[15]	N	Yes	N	N	Yes		104	23
AML47	BM	25	M	Intermediate	46XY [20]	N	N	Yes	Yes	Yes		1.9	20
AML48	BM	34	F	Intermediate	46XX[20]	Yes	N	Yes	N	N		89.3	22

Table S3. AML mutations in our patient population. (A) Prevalence of mutations in our 48 AML samples. (B) Distribution of mutations with ascending age and cytogenetic classification.

FLT3-ITD and NPM1 mutations obtained from clinical information where available. FLT3-ITD typing performed by Levis laboratory at Johns Hopkins University (according to method described in Murphy KM *et al*[1]). Additional mutation data obtained by analyzing DNA from AML samples on qBiomarker Somatic Mutation PCR Array for human acute myeloid leukemia (Qiagen, Valencia, CA) according to manufacturer's instructions.

[1]Murphy KM, Levis M, Hafez MJ, Geiger T, Cooper LC, Smith BD, et al. Detection of FLT3 Internal Tandem Duplication and D835 Mutations by a Multiplex Polymerase Chain Reaction and Capillary Electropheresis Assay. Journal of Molecular Diagnostics 2003 May; 5(2); 96-102

(A)

Mutation	Prevalence
FLT3-ITD	17% (8/48)
FLT3-TKD	10% (5/48)
IDH1	8% (4/48)
IDH2	15% (7/48)
KIT	25% (12/48)
NPM1	21% (10/48)
RUNX1	4% (2/48)
TET2	4% (2/48)
WT1	2% (1/48)

(B)

Patient Code	AML29	AML47	AML6	AML24	AML26	AML3	AML8	AML44	AML4	AML48	AML23	AML2	AML20	AML7	AML22	AML42	AML18	AML10PB	AML46	AML27	AML5	AML14	AML30	AML32
Cytogenetic Classification	I	I	I	I	I	F	F	F	I	I	F	A	F	I	I	I	I	F	A	A	I	I	I	A
FLT3-ITD				X						X	X			X	X		X						X	
FLT3-TKD								X				X				X			X					
IDH1		X														X					X			
IDH2		X																	X					
KIT	X							X		X				X					X					X
NPM1		X								X					X						X			
RUNX1																								
TET2																				X				
WT1																			X					
Age	24	25	30	31	31	32	32	33	34	34	36	39	39	39	40	42	44	48	50	51	51	52	55	56

Patient Code	AML35	AML21	AML25	AML28	AML15	AML17	AML12PB	AML34	AML16	AML33	AML13	AML11PB	AML9PB	AML1	AML45	AML37	AML41	AML38	AML36	AML19	AML40	AML31	AML43	AML39
Cytogenetic Classification	A	F	I	I	I	I	I	I	I	I	A	I	I	A	A	A	A	I	I	A	I	I	I	I
FLT3-ITD																					X			
FLT3-TKD																		X						
IDH1								X																
IDH2										X							X	X	X				X	
KIT			X					X	X		X	X										X		
NPM1				X	X			X				X								X				X
RUNX1			X																X					
TET2												X												
WT1																								
Age	56	56	57	57	58	59	60	60	61	61	65	65	65	67	67	68	68	68	69	70	73	76	78	86

Figure Legend

Cytogenetic Classification

Cytogenetic Classification
FAVORABLE
INTERMEDIATE
ADVERSE

Table S4. RNA integrity numbers (RIN). (A) RINs of our 48 patients, healthy donor PB and BM, normal organ tissues, and K562 cell line. (B) Graphs of ribosomal peaks with RINs of 10, 7, 5.5, and 2.8. Based on integrity of ribosomal peaks, we used only samples with RINs ≥ 7 for reliable gene expression output.

(A)

Patient Code	Sample Type	RIN
AML1	PB	9.3
AML2	PB	9.0
AML3	PB	7.9
AML4	PB	9.5
AML5	PB	8.6
AML6	PB	7.6
AML7	PB	10.0
AML8	PB	7.2
AML9PB	PB	8.1
AML10PB	PB	9.7
AML11PB	PB	7.1
AML12PB	PB	8.1
AML13	PB	7.4
AML14	PB	7.5
AML15	PB	9.0
AML16	PB	9.6
AML17	PB	7.0
AML18	PB	7.3
AML19	PB	7.1
AML20	PB	8.9
AML21	PB	7.2
AML22	PB	8.7
AML23	PB	9.8
AML24	PB	7.0
AML25	PB	9.6
AML26	PB	8.8
AML27	PB	7.9
AML28	PB	7.4
AML29	PB	7.4
AML30	PB	7.2
AML9BM	BM	9.1
AML10BM	BM	8.5
AML11BM	BM	7.9
AML12BM	BM	7.6
AML31	BM	8.7
AML32	BM	7.4
AML33	BM	7.5
AML34	BM	8.5
AML35	BM	8.3
AML36	BM	9.7
AML37	BM	7.2
AML38	BM	7.6
AML39	BM	9.2
AML40	BM	7.9
AML41	BM	9.3
AML42	BM	7.8
AML43	BM	7.4
AML44	BM	7.0
AML45	BM	9.5
AML46	BM	9.2
AML47	BM	8.6
AML48	BM	10.0
NPB1	PB	10.0
NPB2	PB	9.8
NPB3	PB	10.0
NPB4	PB	9.0
NPB5	PB	9.8
NPB6	PB	9.9
NPB7	PB	10.0
NPB8	PB	9.8
NPB9	PB	10.0
NPB10	PB	10.0
NBM1	BM	7.0
NBM2	BM	7.0
NBM3	BM	9.6
NBM4	BM	7.6
NBM5	BM	8.1
NBM6	BM	8.2
NBM7	BM	8.5
Whole Brain	Tissue	7.8
Lung	Tissue	7.5
Liver	Tissue	7.4
Heart	Tissue	7.8
Cerebellum	Tissue	8.2
Thyroid	Tissue	7.1
Colon	Tissue	8.0
Prostate	Tissue	7.5
Muscle	Tissue	7.7
Intestine	Tissue	6.9
Kidney	Tissue	8.9
Spleen	Tissue	7.2
K562	Cell Line	10.0

(B)

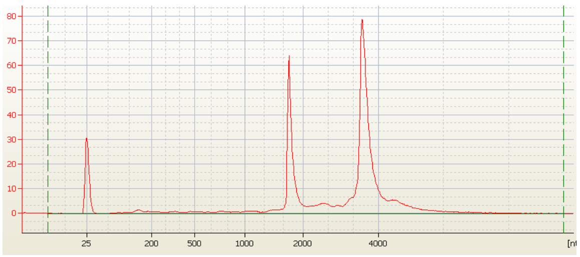

RIN = 10.0

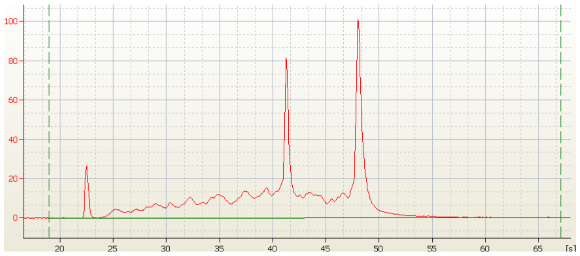

RIN = 7.0

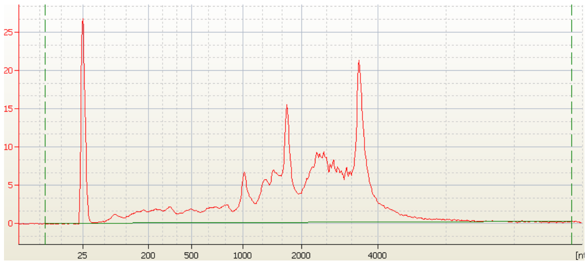

RIN = 5.5

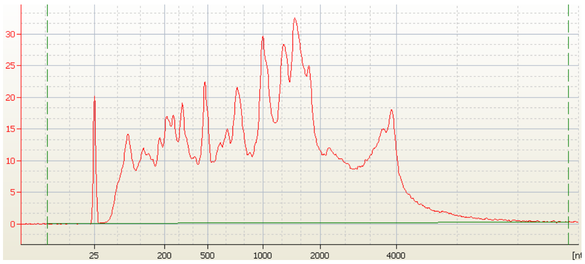

RIN = 2.8

Table S5. Normal organ tissue information.

Clontech
Human Total RNA Master Panel II
Catalog Number: 636643
Lot Number: 1112058A

Organ RNA	*Source*
Brain (whole)	43-year-old male Caucasian
Lung	pooled from 3 male/female Caucasians, ages: 32-61
Liver	51-year-old male Caucasian
Heart	pooled from 3 male Caucasians; ages: 30-39
Cerebellum	pooled from 10 male/female Caucasians, ages: 22-68
Thyroid gland	pooled from 64 male/female Caucasians; ages 15-61
Prostate	pooled from 12 Caucasians; ages 20-58
Skeletal Muscle	pooled from 2 male/female Caucasians; ages 43-63
Small Intestine	pooled from 5 male/female Caucasians; ages 20-61
Kidney	40-year-old female Caucasian
Spleen	pooled from 15 male/female Caucasians; ages 22-69

All information taken from Clontech certificate of analysis dated 2/2/12

Clontech
Human Colon Total RNA
Catalong Number: 636553
Lot Number: 1005014

Normal human colon with mucosal lining	pooled from 5 male Asians; ages: 20-44, cause of death: sudden death

All information taken from Clontech certificate of analysis

Table S6. Antigen expression in sorted leukemia cell populations of 8 AML patients.

Gene	AML 14				AML 23				AML 24				AML 25				AML 27				AML 28				AML 29				AML 30			
	U	B	N	L	U	B	N	L	U	B	N	L	U	B	N	L	U	B	N	L	U	B	N	L	U	B	N	L	U	B	N	L
PRTN3	-	-	-	++	++	+	+	++	+	+	-	+	+	+	-	+++	+++	++	+	++	++	-	-	++	-	+	++	+++	-	+++	+	+++
WT1	+	+	-	++	++	++	++	++	++	++	-	+					++	++	++	+++					+	++	-	++	+++	+++	-	-
HOXA9									++	++	-	++									++	++	-	+	-	-	-	++	+++	+++	-	+++
BAALC									+	+	+	++					+	-	-	++									++	-	-	+++
CCNA1					+++	++	++	++	++	++	-	-					++	++	+	++												
GAGE1	-	-	-	++																									-	-	-	++
KIT	+	+	-	++																									++	++	-	++
MEIS1																					+	+	-	+					++	++	-	++
ERG					+	+	+	+	++	++	-	++																				
HOXB4																													++	++	-	++
HBG2	+++	+++	-	+++																												
CD276					+	+	+	+																								
PRAME																	++	++	-	+++												

Figure Legends

Leukemia Cell Populations

U	Unsorted bulk leukemia
B	Leukemic blast population
N	Non-leukemia population
L	CD34+CD38-Lin- enriched stem cell population

Antigen expression in cell populations

- Only expressed in stem cell
- Only expressed in bulk leuk
- Highly expressed in stem cell.
- Homogenous thru all leukemia populations
- Expressed in unsorted and stem cell

Magnitude of over-expression (OE)

+	10-50x OE
++	50-500x OE
+++	<500x OE

Figure S1. Expression of leukemia-associated antigens (LAA) in K562. (A) Expression values from our custom array are highly reproducible. K562 was analyzed twice using different plates on different days. Antigen expression is highly similar between both runs. (B) Heat map of LAA expression in K562 normalized to median expression seen in healthy peripheral blood (PB). Light red indicates over-expression (OE) of 5-50x, red indicates OE of 50-500x, and bright red indicates OE of greater than 500x. K562 is a good positive control for most genes not expressed in AML PB or healthy PB.

Figure S2. Antigen expression in FLT3-ITD mutated AML. (A) RHAMM, PR3, Survivin, and PRAME are not associated with FLT3-ITD mutation status. (B) WT1 is strongly associated with FLT3-ITD mutation status.

Figure S3. RNA Integrity (RIN) versus expression of common housekeeping genes. Samples with lower RIN values trend towards higher C(t) values for these genes.

Figure S4. Peripheral blood (PB) versus bone marrow (BM) from the same patient. In 3/4 patients, PB showed same expression profile as BM; patient 12 had differing expression gene expression values for several genes between PB and BM.

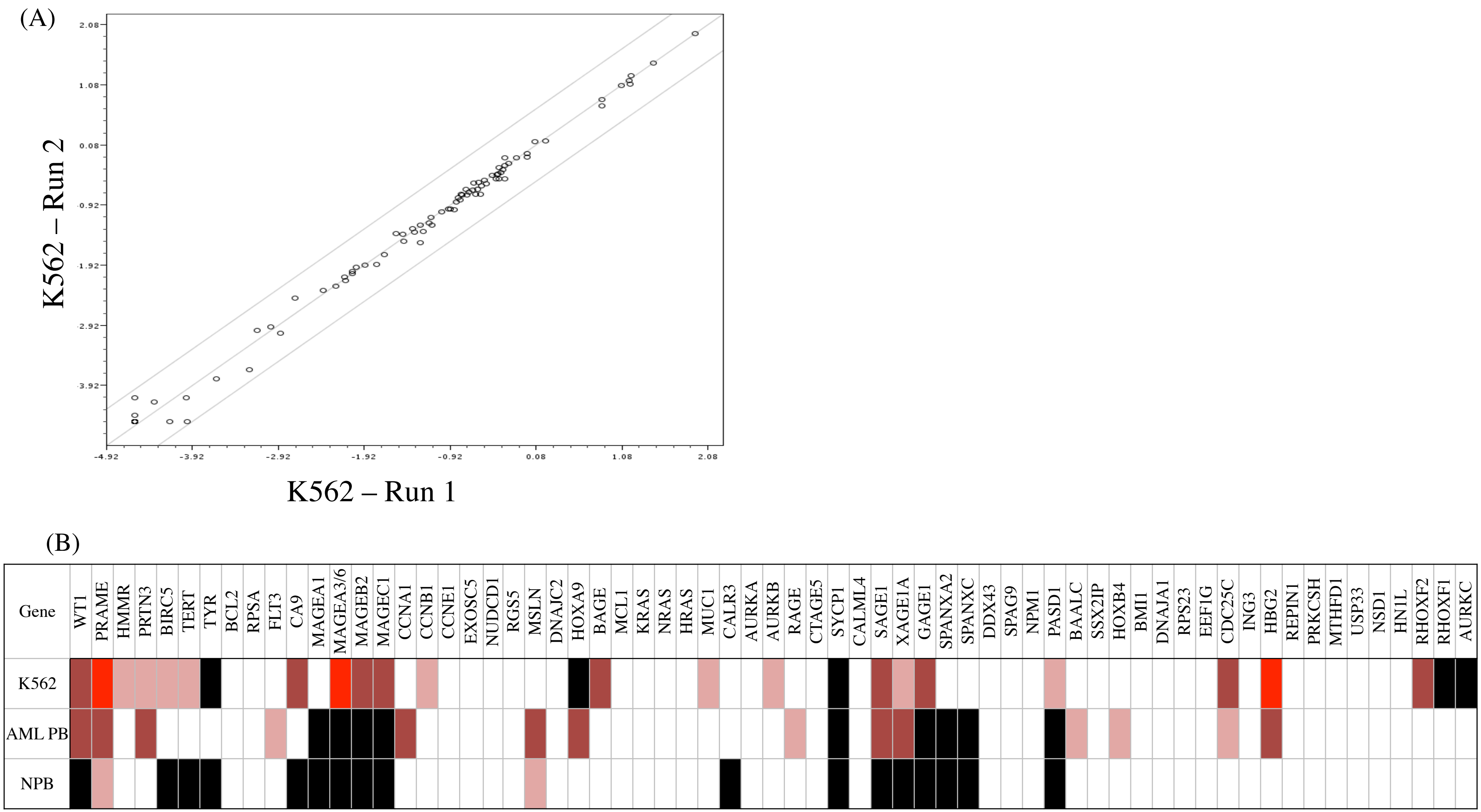
(A)
K562 – Run 2
K562 – Run 1
2.08
1.08
0.08
-0.92
-1.92
-2.92
-3.92
-4.92
(B)
Gene
WT1 PRAME HMMR PRTN3 BIRC5 TERT TYR BCL2 RPSA FLT3 CA9 MAGEA1 MAGEA3/6 MAGEB2 MAGEC1 CCNA1 CCNB1 CCNE1 EXOSC5 NUDCD1 RGS5 MSLN DNAJC2 HOXA9 BAGE MCL1 KRAS NRAS HRAS MUC1 CALR3 AURKA AURKB RAGE CTAGE5 SYCP1 CALML4 SAGE1 XAGE1A GAGE1 SPANXA2 SPANXC DDX43 SPAG9 NPM1 PASD1 BAALC SSX2IP HOXB4 BMI1 DNAJA1 RPS23 EEF1G CDC25C ING3 HBG2 REPIN1 PRKCSH MTHFD1 USP33 NSD1 HN1L RHOXF2 RHOXF1 AURKC
K562
AML PB
NPB

Figure S1

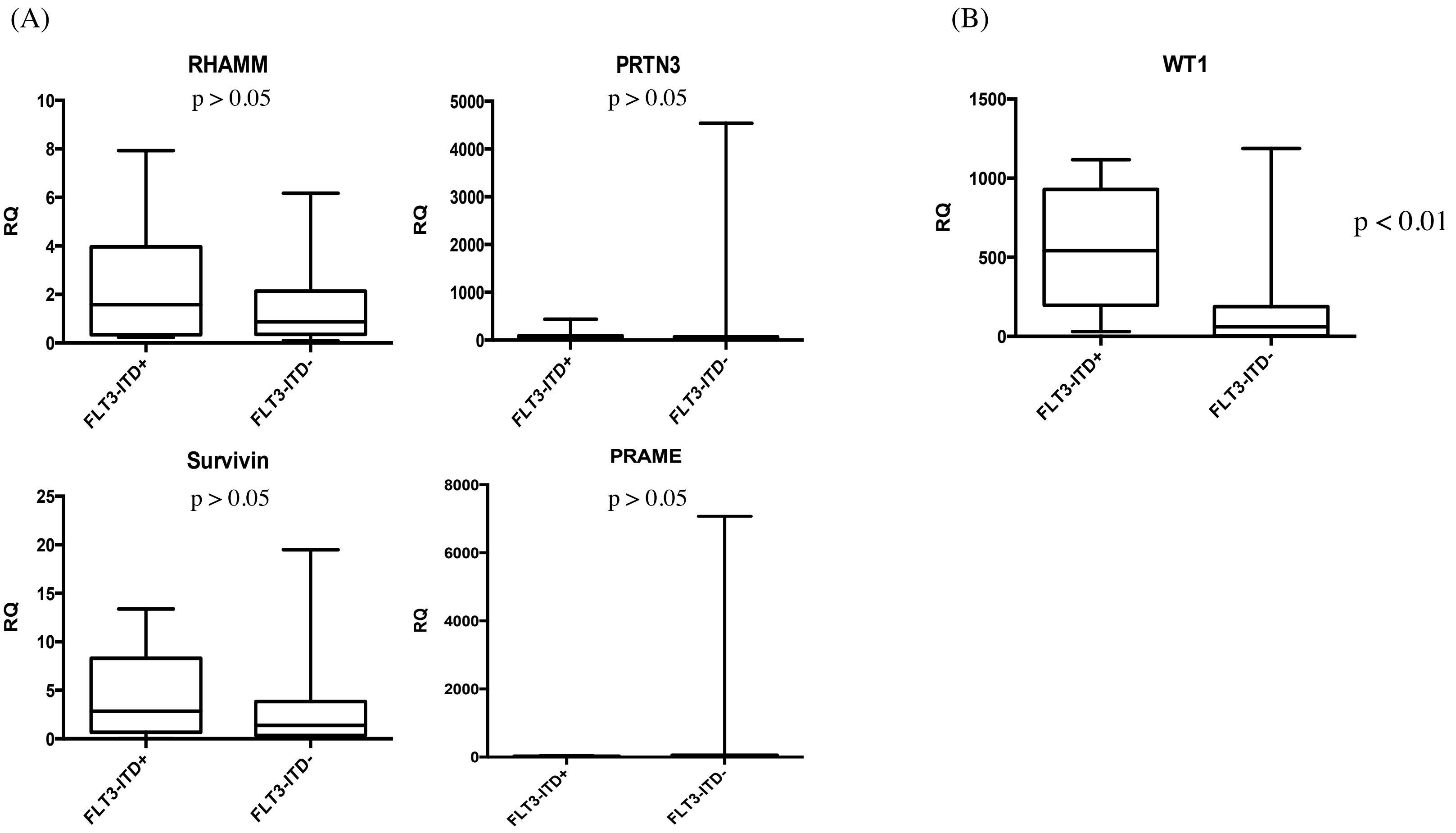
(A)
RHAMM
p > 0.05
RQ
0
2
4
6
8
10
FLT3-ITD+
FLT3-ITD-
PRTN3
p > 0.05
RQ
0
1000
2000
3000
4000
5000
FLT3-ITD+
FLT3-ITD-
Survivin
p > 0.05
RQ
0
5
10
15
20
25
FLT3-ITD+
FLT3-ITD-
PRAME
p > 0.05
RQ
0
2000
4000
6000
8000
FLT3-ITD+
FLT3-ITD-
(B)
WT1
RQ
0
500
1000
1500
p < 0.01
FLT3-ITD+
FLT3-ITD-

Figure S2

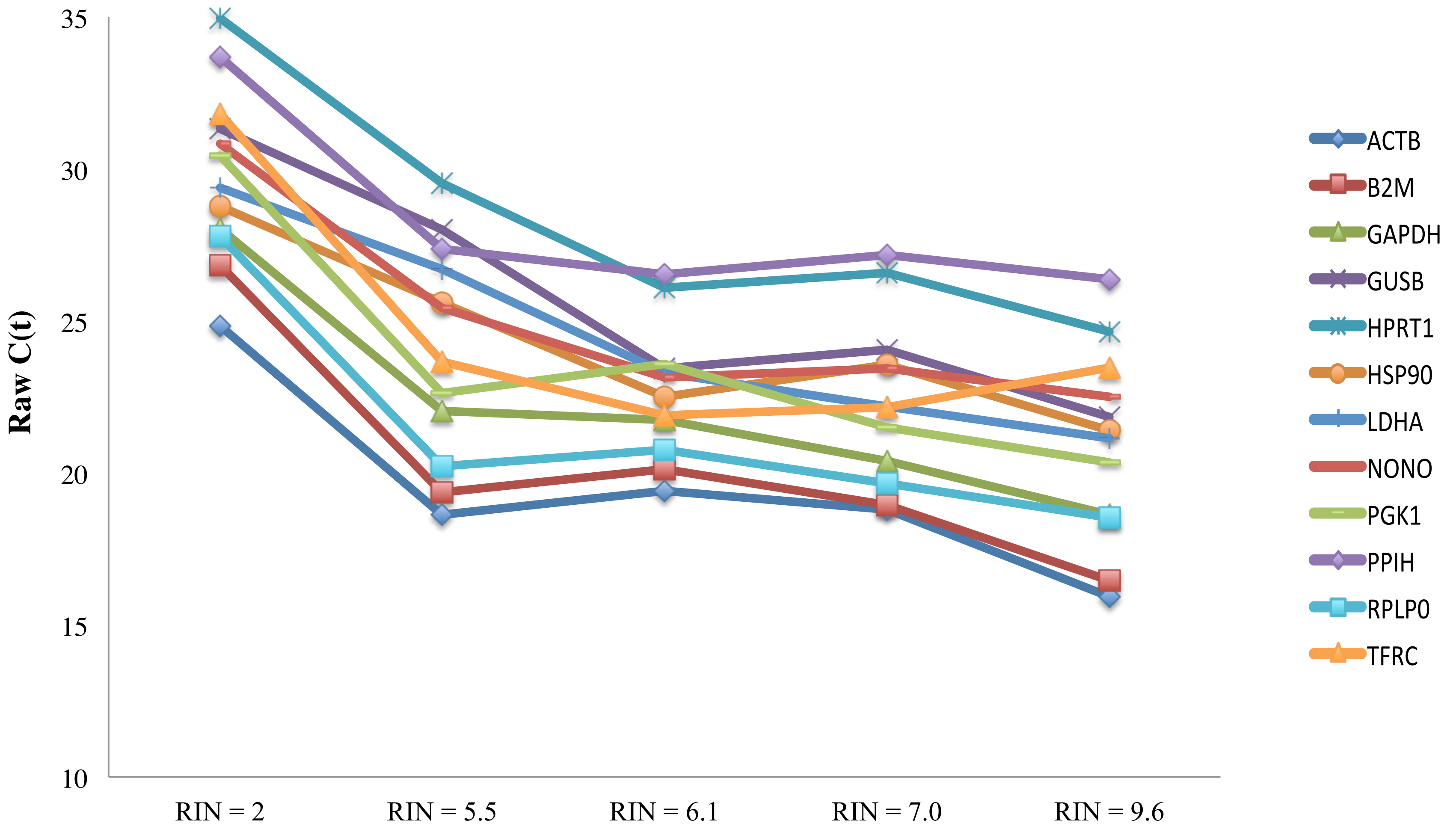
35
30
25
20
15
10
Raw C(t)
RIN = 2
RIN = 5.5
RIN = 6.1
RIN = 7.0
RIN = 9.6
ACTB
B2M
GAPDH
GUSB
HPRT1
HSP90
LDHA
NONO
PGK1
PPIH
RPLP0
TFRC

Figure S3

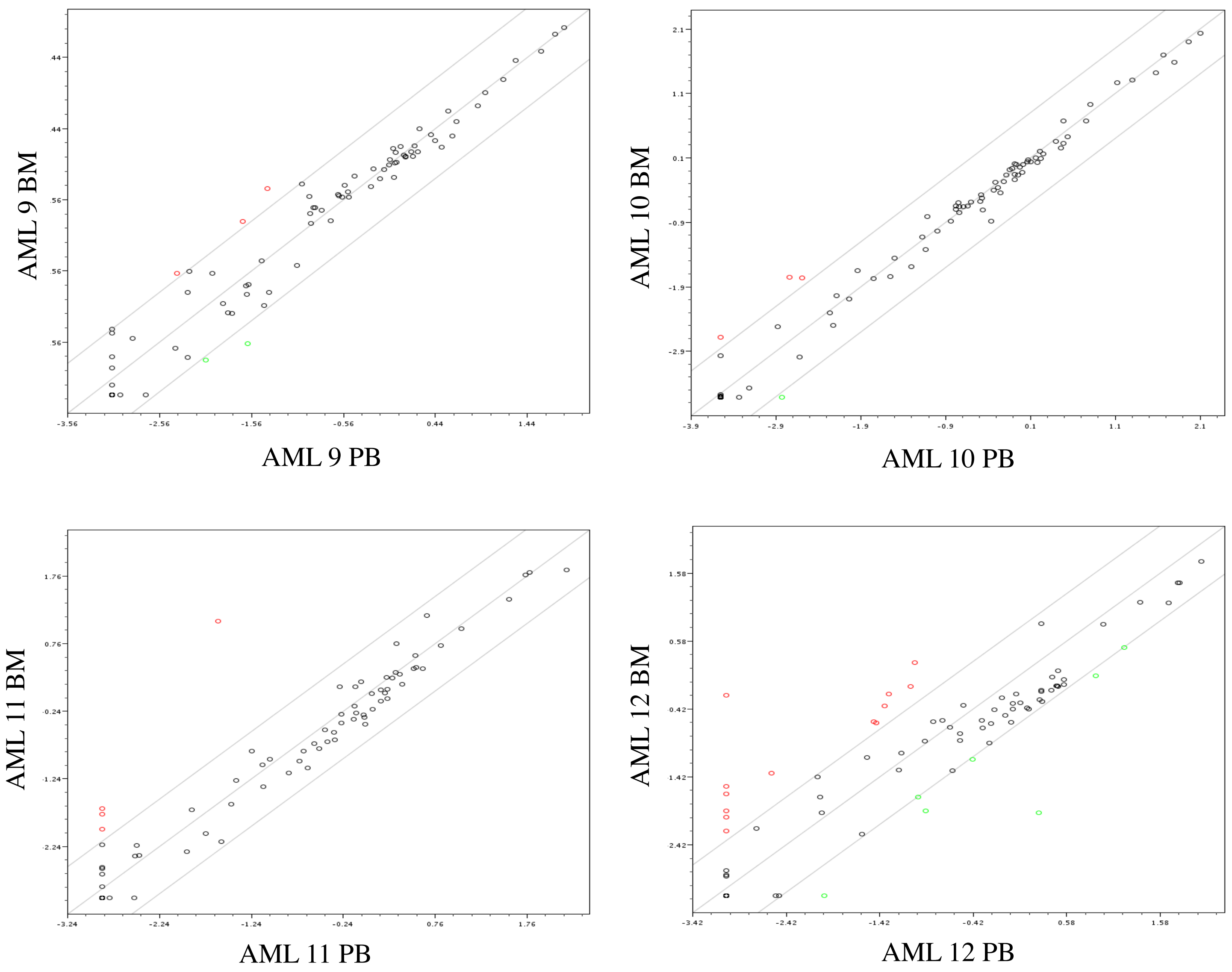
AML 9 BM
-3.56
-2.56
-1.56
-0.56
0.44
1.44
AML 9 PB
AML 10 BM
2.1
1.1
0.1
-0.9
-1.9
-2.9
-3.9
AML 10 PB
AML 11 BM
1.76
0.76
-0.24
-1.24
-2.24
-3.24
AML 11 PB
AML 12 BM
-3.42
-2.42
-1.42
-0.42
0.58
1.58
AML 12 PB

Figure S4
